# Quantification of Hsp90 availability reveals differential coupling to the heat shock response

**DOI:** 10.1083/jcb.201803127

**Published:** 2018-11-05

**Authors:** Brian D. Alford, Onn Brandman

**Affiliations:** Department of Biochemistry, Stanford University, Stanford, CA

## Abstract

The heat shock response (HSR) is a protective gene expression program that is activated by conditions that cause proteotoxic stress. While it has been suggested that the availability of free chaperones regulates the HSR, chaperone availability and the HSR have never been precisely quantified in tandem under stress conditions. Thus, how the availability of chaperones changes in stress conditions and the extent to which these changes drive the HSR are unknown. In this study, we quantified Hsp90 chaperone availability and the HSR under multiple stressors. We show that Hsp90-dependent and -independent pathways both regulate the HSR, and the contribution of each pathway varies greatly depending on the stressor. Moreover, stressors that regulate the HSR independently of Hsp90 availability do so through the Hsp70 chaperone. Thus, the HSR responds to diverse defects in protein quality by monitoring the state of multiple chaperone systems independently.

## Abstract (precis)

Alford and Brandman quantify the ability of the Hsp90 chaperone system to fold its client proteins and describe how loss of this functionality affects the heat shock response. They find that the heat shock response responds to diverse defects in protein quality by monitoring the state of multiple chaperone systems independently.

## Introduction

The heat shock response (HSR) is a highly conserved transcriptional program that defends against stress caused by the accumulation of misfolded protein (proteotoxic stress; Åkerfelt et al., 2007; Morimoto, 2011). Proper HSR levels are critical for the normal clearance of ubiquitinated proteins as well as surviving toxic stressors such as heat and oxidative damage (Morano et al., 2012). Modulating the HSR is emerging as a promising treatment for neurodegeneration and HSR-dependent cancers (Whitesell and Lindquist, 2009; Neef et al., 2010). Therefore, understanding the mechanisms driving the HSR is of great importance for understanding fundamental biology, disease, and design of protective therapies.

Innovative and insightful biochemical studies have demonstrated that the transcription factor that drives the HSR, Hsf1, undergoes an immense number of changes in response to stress; Hsf1 is phosphorylated, sumoylated, acetylated, and degraded and undergoes large changes in conformation and binding partners (Xia and Voellmy, 1997; Bonner et al., 2000; Hietakangas et al., 2003; Westerheide et al., 2009). This complexity has led to a correspondingly vast number of models for HSR activation (Morimoto, 1998; Shamovsky and Nudler, 2009; Anckar and Sistonen, 2011). Yet despite these efforts, understanding which regulatory mechanisms drive the HSR in the context of stress, health, and disease remains elusive.

**Figure 1.**
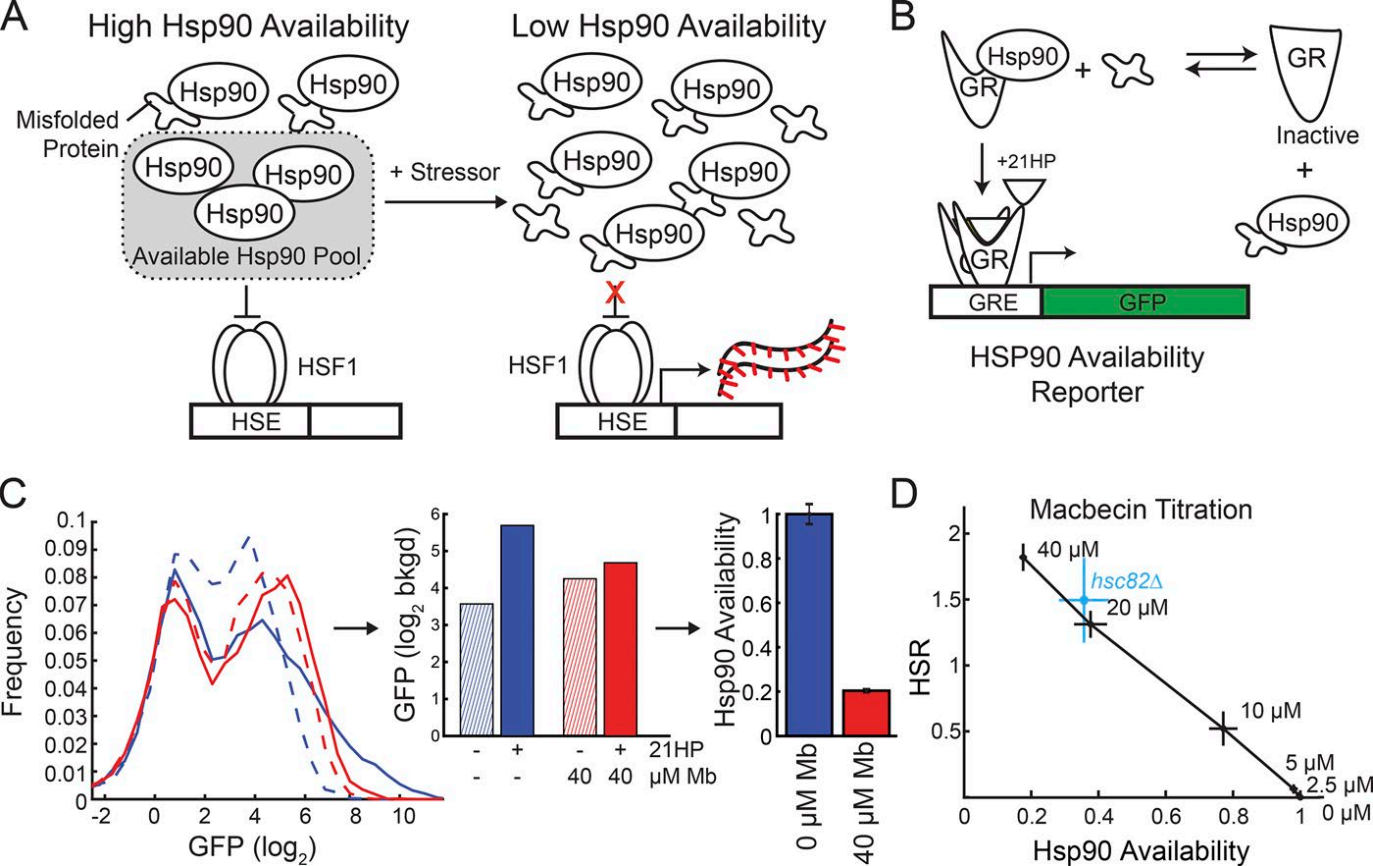
**Measuring Hsp90 availability using a quantitative fluorescent reporter. (A)** Schematic of Hsp90 availability model of HSR regulation. **(B)** Schematic of Hsp90 availability reporter. **(C)** Left: Histogram of forward scatter–normalized GFP fluorescence either with 0 µM (dashed lines) or 10 µM (solid lines) 21HP and treatment with either 0 µM (blue) or 40 µM (red) macbecin (Mb). Middle: GFP fluorescence from each condition was used to quantify GR activity. bkgd, background. Right: Difference in GR activity with and without 21HP was used to calculate Hsp90 availability. **(D)** Plot of HSR versus Hsp90 availability for a titration of macbecin. *n* > 3; mean ± SEM.

One prevalent class of models posits that the HSR senses misfolded protein levels through changes in chaperone availability (Fig. 1 A; Craig and Gross, 1991; Abravaya et al., 1992). In these models, the HSR is basally inhibited by chaperone folding activity. Under proteotoxic stress conditions, misfolded proteins consume available chaperone capacity, thus reducing the pool of available chaperones to inhibit the HSR. Hsp90 (Nadeau et al., 1993; Ali et al., 1998; Zou et al., 1998), Hsp70 (Bonner et al., 2000; Zheng et al., 2016), and Hsp60 (Neef et al., 2014) have been reported to bind to Hsf1. This has led to speculation that loss of the chaperone–Hsf1 interaction is the regulatory step by which proteotoxic stressors activate the HSR. Yet maintenance of chaperone–client interactions is critical for the function of numerous client proteins such as steroid receptors (Zhang and Burrows, 2004) but are not their primary mechanism of regulation. Thus, rather than drive the HSR, changes in chaperone–Hsf1 affinity could be ancillary to other mechanisms of HSR regulation.

To demonstrate a causal relationship between chaperone availability and the HSR for a given stressor, two systems-level features must be quantified: (1) the relationship between direct changes to chaperone availability and HSR levels and (2) the change in chaperone availability and HSR activity under the stressor. With these two quantities, the extent to which HSR activation can be attributed to direct reduction in chaperone availability can be determined. To generate these data, we developed a fluorescent Hsp90 availability reporter and made quantitative measurements of Hsp90 chaperone availability and the HSR in the budding yeast *Saccharomyces cerevisiae* under diverse proteotoxic stress conditions. This allowed us to determine for the first time how several proteotoxic stresses affect Hsp90 availability and whether this explains HSR activity. We combined this analysis with epistasis experiments to elucidate how the Hsp70 and Hsp90 chaperone systems regulate the HSR. Our work provides a framework for using quantitative readouts of quality control systems to establish causative relationships between chaperones and their downstream processes.

## Results and discussion

### Fluorescent reporters quantitatively measure Hsp90 availability and HSR activation

To understand the relationship between Hsp90 availability and the HSR, we first needed a quantitative reporter to measure levels of Hsp90 availability. Chaperone availability is qualitatively defined as the level of free chaperones available to bind unbound client proteins. If misfolded protein increases such that the levels of available chaperones become limiting, a greater fraction of that chaperone clients will be unbound and consequently misfolded. Our strategy to quantify Hsp90 availability was thus to measure the folding state of a well-studied client protein of Hsp90, glucocorticoid receptor (GR; Scherrer et al., 1990). Though not an endogenous yeast protein, the mammalian gene has been demonstrated to retain its Hsp90-dependent activity in yeast cells (Kimura et al., 1995). We therefore developed an Hsp90 availability reporter system consisting of mammalian GR expressed in yeast along with a plasmid containing a synthetic GR-binding promoter driving GFP expression (Fig. 1 B).

Addition of the glucocorticoid 21-hydroxyprogesterone (21HP) caused a 4.3-fold (2.1 log_2_ units) induction of GFP fluorescence to cells containing our Hsp90 reporter (Fig. 1 C, blue data). We defined the GR induction in untreated, WT cells to be one unit of Hsp90 availability by adjusting the base of the logarithm (see Materials and methods). To test the dependence of GFP induction on Hsp90 availability, we treated cells with the Hsp90 inhibitor macbecin (Martin et al., 2008). Macbecin-treated cells showed a marked decreased in GFP induction (0.2 U of Hsp90 availability), demonstrating the reporter’s sensitivity to changes in Hsp90 capacity (Fig. 1 C, red data).

To measure the HSR, we used a previously developed reporter containing a synthetic Hsf1-dependent promoter driving GFP expression (Brandman et al., 2012). When macbecin was added to cells containing the reporter, the HSR was activated, and GFP levels increased. This confirms that Hsp90 inhibition alone is sufficient to activate the HSR. We used the macbecin titration for these two quantitative reporters to build a profile of the expected HSR levels for a given amount of Hsp90 availability (Fig. 1 D). The increase in HSR activity was concomitant with the changes in Hsp90 availability, suggesting that the HSR is not affected until the buffering pool is exhausted. Additionally, the deletion of one of two copies of Hsp90 (*hsc82*Δ) mimicked ∼20 µM macbecin, showing that the effects of macbecin are specific to Hsp90 and not from off-target activity. From these data, we concluded that our reporter systems provide robust and quantitative measurements of Hsp90 availability and the HSR in vivo.

### Different proteotoxic stresses have distinct effects on Hsp90 availability

We hypothesized that it was possible that different methods of creating misfolded protein could have different effects on the client load for cellular chaperone systems. We used seven diverse mechanisms of inducing proteotoxic stress conditions consisting of macbecin, which inhibits Hsp90, bortezomib, which inhibits the proteasome, canavanine (Can) and azetidine-2-carboxylic acid (AZC), which misincorporate into nascent chains in the place of arginine and proline, respectively (Rodgers and Shiozawa, 2008), arsenite, which binds to sulfhydryl groups (Shen et al., 2013), ethanol, which disrupts hydrophobic interactions (Brandts and Hunt, 1967), and heat, which causes thermal denaturation of proteins.

We first measured cell viability and protein expression to identify the appropriate stressor concentration ranges in which our fluorescence-based reporter systems were still effective. The concentrations of drugs that we used did not cause cell death (Fig. S1 A) and allowed gene expression from at least one of two promoters (a constitutive promoter from the *TDH3* gene and a *GAL4*-inducible promoter; Fig. S1, B and C). Additionally, we verified that changes in Hsp90 availability reporter mRNA correlated with changes in the reporter’s protein production (Fig. S1, D and E). Thus, we concluded that changes in our fluorescent reporter systems cannot be explained by cell death or general defects in gene expression and are instead a consequence of changes in Hsp90 availability and the HSR.

**Figure 2.**
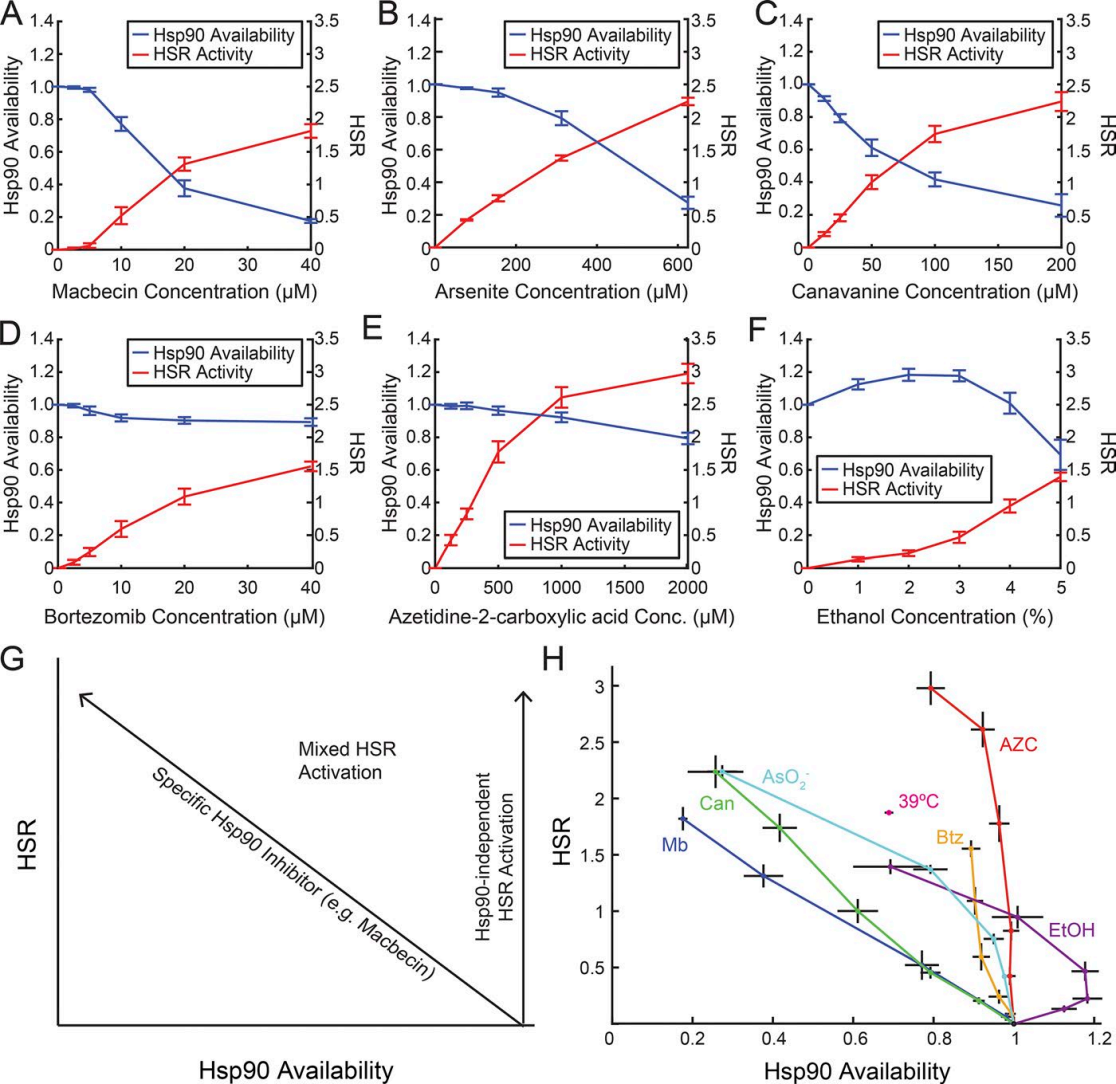
**Proteotoxic stresses have diverse effects on Hsp90 availability and HSR activity. (A–F)** Measurements of Hsp90 availability (blue line starting at 1 U) and HSR activity (red line starting at 0 U) in titrations of various proteotoxic stressors. **(G)** Model for quantifying contribution of changes in Hsp90 availability with changes in the HSR. **(H)** HSR versus Hsp90 availability in the environmental stressors shown in A–F as well as in 39°C heat shock (39°C; magenta). Mb, macbecin. *n* > 3; mean ± SEM.

All of the stresses we tested robustly activated the HSR (Fig. 2, A–F; red lines), yet diverse effects on Hsp90 availability were observed (Fig. 2, A–F; blue lines). Similar to the Hsp90 inhibitor macbecin (Fig. 2 A), addition of arsenite (Fig. 2 B) and Can (Fig. 2 C) caused a robust decrease in Hsp90 availability. Other stresses had much milder effects on Hsp90 availability. Bortezomib caused a minor decrease (Fig. 2 D), while AZC caused virtually no change in Hsp90 availability (Fig. 2 E). Ethanol had an unusual effect, causing a small increase in Hsp90 availability at low ethanol concentrations followed by a sharp decrease at higher concentrations (Fig. 2 F). The varied effects on the Hsp90 system suggest that different forms of proteotoxic stress (as measured by HSR activation) have different effects on the chaperone network.

### The HSR has both Hsp90-dependent and Hsp90-independent methods of activation

We next determined how Hsp90 availability was related to the HSR under diverse stressors. Our measurements of Hsp90 and the HSR allowed us distinguish between three regimes (Fig. 2 G). Stresses that activate the HSR solely by burdening the Hsp90 chaperone system (Hsp90-dependent HSR activation) are predicted to show the same relationship between Hsp90 availability and HSR activation as macbecin. By contrast, stresses that activate the HSR without reducing Hsp90 availability activate the HSR through Hsp90-independent mechanisms. Finally, stressors may drive the HSR through a mixture of these modes of activation.

Strikingly, the stresses we tested caused HSR activation in all three regimes (Fig. 2 H). AZC in particular induced a large HSR without substantially decreasing Hsp90 availability (Hsp90-independent HSR activation). Bortezomib and low concentrations of ethanol also primarily induced HSR without large changes to Hsp90 availability. By contrast, low amounts of Can and high amounts of Ars caused almost completely Hsp90-dependent HSR activation. Finally, low amounts of arsenite and Can, high levels of ethanol, and 39°C heat shock exerted their effect on the HSR activation through a mixture of Hsp90-dependent and -independent mechanisms. We conclude that changes in Hsp90 availability explain the majority of HSR activation under some stresses (Hsp90-dependent HSR activation) but are not sufficient to explain the HSR induced by other stress conditions, and thus, in these cases, HSR must be modulated by other factors (Hsp90-independent HSR activation).

As a possible mechanism by which stressors regulate Hsp90 availability, we measured Hsp90 levels in each stress condition (Fig. S2). Hsp90 levels increased substantially in macbecin and AZC, were unchanged in Can and arsenite, and decreased in ethanol. This observation may explain some of the differences between AZC and Can, which have substantially different effects on Hsp90 availability despite acting through similar mechanisms. However, it is important to note that low levels of AZC do not cause significant changes in Hsp90 levels yet still activate the HSR in an Hsp90-independent manner. Additionally, Hsp90 levels did not correlate with Hsp90 availability in any of the other stressors, suggesting that Hsp90 levels are not the single determinative factor Hsp90 availability and that Hsp90 levels are regulated in an Hsf1-independent manner.

### Multiple protein quality control systems regulate the HSR independently of Hsp90

Having found that exogenous stressors activate the HSR through both Hsp90-dependent and -independent mechanisms, we then sought to evaluate how genetic disruption of cellular quality control pathways affects Hsp90 availability. We focused on quality control genes that were known to robustly activate the HSR when deleted (Brandman et al., 2012). These genes coded for components of the Hsp90 chaperone system (*HSC82* and *STI1*; Richter et al., 2003), the Hsp70 chaperone system (*SSA2* and *STI1*; Wegele et al., 2003), the Hsp104 chaperone (*HSP104*), the tail-anchored protein trafficking system for the ER (*GET3*; Schuldiner et al., 2008), a cotranslational protein quality control system (*RQC1* and *LTN1*; Brandman et al., 2012), and proteins with unknown functions that clustered with Hsp90 in a genetic interaction map (*YPL225W*, *HGH1*, and *AIM29*; Brandman et al., 2012).

**Figure 3.**
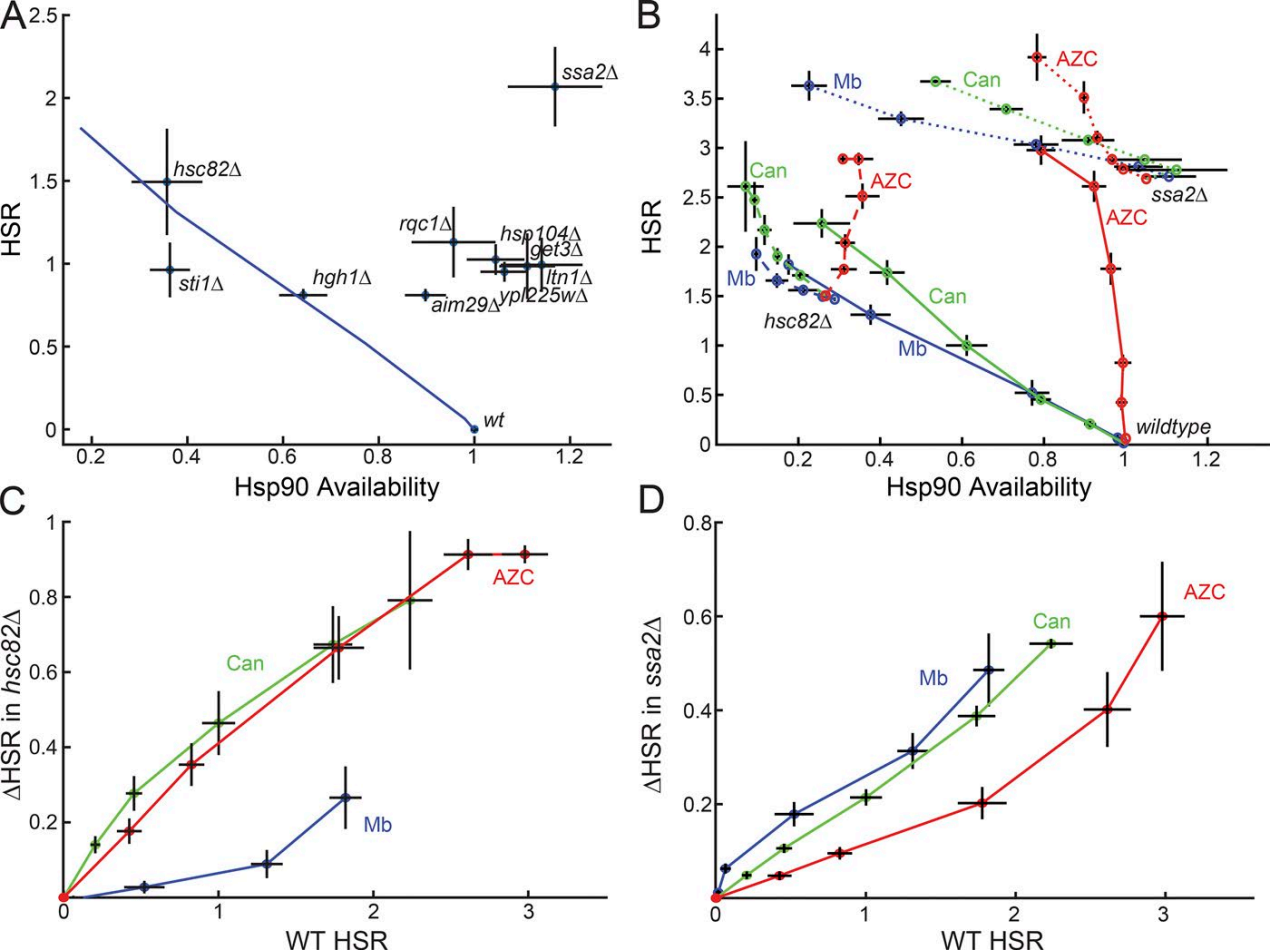
**The HSR is activated independently by the Hsp90 and Hsp70 chaperone systems. (A)** HSR versus Hsp90 availability for indicated gene deletions. Macbecin titration (blue) included as a reference for Hsp90-dependent HSR activation. **(B)** The HSR versus Hsp90 availability in titrations of macbecin (blue), Can (green), and AZC (red) in *hsc82Δ* cells (dashed lines) and *ssa2Δ* cells (dotted lines). WT titrations from Fig. 2 (solid) are included for reference. **(C)** Change in the HSR for each of the three stressors in WT versus *hsc82Δ* cells. **(D)** Change in the HSR for each of the three stressors in WT versus *ssa2Δ* cells. *n* > 3; mean ± SEM.

The *get3Δ*, *hsp104Δ*, *rqc1Δ*, and *ltn1Δ* strains all exhibited a moderately increased HSR without substantial changes in Hsp90 availability. Strikingly, depleting the Hsp70 paralog Ssa2 caused the strongest activation of the HSR and a concomitant increase in Hsp90 availability (Fig. 3 A). This was particularly interesting since the deletion of SSA2 alone is typically not a strong enough perturbation to significantly impact the performance of the Hsp70 system (Park et al., 2007; Maurer et al., 2016). Thus, these quality control systems demonstrate mostly Hsp90-independent HSR activation.

By contrast, the few proteins that were found to activate the HSR while also affecting Hsp90 availability appeared to be closely related to Hsp90. Deleting *HSC82* (one of two genes encoding Hsp90) or the Hsp90 cochaperone *STI1* caused large decreases in Hsp90 availability as well as robust activation of the HSR (Fig. 3 A). Of the three genes of unknown function that clustered with Hsp90 in an interaction map from a previous study (Brandman et al., 2012), only Hgh1 substantially impaired Hsp90 availability. Given that it is one of the few genetic perturbations we tested that affected Hsp90 availability, we speculate that Hgh1 may be a direct regulator of Hsp90. Consistent with this hypothesis, three high throughput studies found that Hgh1 physically associates with Cns1 (Gavin et al., 2002; Tarassov et al., 2008; Schlecht et al., 2012), a conserved Hsp90 and Hsp70 cochaperone.

### The HSR senses proteotoxic stress through Hsp90 and Hsp70 independently

The strongest activator of the HSR from our panel of deletions was of an Hsp70 chaperone, *SSA2*. Additionally, the Ssa proteins have been demonstrated to dynamically alter binding to Hsf1 under stress (Zheng et al., 2016; Krakowiak et al., 2018). We therefore investigated whether the HSR sensed proteotoxic stress through a loss of Hsp70 availability. Since we lacked a suitable substrate to measure Hsp70 availability, we instead used an epistasis analysis strategy. We added proteotoxic stresses to yeast with impaired Hsp70 function and determined how Hsp70 depletion affected HSR activation. We reasoned that if proteotoxic stress signaled through loss of Hsp70 availability, then deleting a copy of the Hsp70 chaperone would reduce HSR induction if that induction is Hsp70 dependent. We chose macbecin, Can, and AZC, which caused complete, partial, and no Hsp90-dependent HSR activation, respectively. As with the WT strain, we verified that general gene expression was not impaired in Hsp90 and Hsp70 deletion strains through measuring viability, protein production, and galactose-induced protein production (Fig. S3, A–C).

To test this epistasis strategy, we first evaluated how these stressors interacted with a yeast strain with an impaired Hsp90 system (*hsc82Δ*). Adding macbecin to the *hsc82*Δ strain resulted in only a minor increase in HSR activation, whereas addition of Can or AZC robustly increased the HSR in the *hsc82*Δ background (Fig. 3 B). We then quantified the effect of Hsp90 impairment by comparing stress-induced changes in the HSR observed in the WT and *hsc82*Δ backgrounds (Fig. 3 C). Consistent with our model, HSR activation by macbecin was dramatically impaired by deletion of Hsp90, while HSR activation by Can and AZC was preserved.

We next evaluated the role of Hsp70 in sensing proteotoxic stress using the epistasis strategy by stressing cells in the Hsp70-impaired background (*ssa2*Δ). Deletion of the Hsp70 copy did not substantially change the sensitivity of the Hsp90 system to each stressor (Fig. 3 B). Strikingly, the strongest activator of the HSR in the Hsp70-impaired strain was the most Hsp90-dependent stressor, macbecin, followed by Can and then AZC (Fig. 3 D). Because the amount of Hsp90 independence correlated with the reductions in the HSR when the Hsp70 system was impaired, we hypothesize that the Hsp90-independent stresses are driving HSR activation by reducing Hsp70 availability. Additionally, since inhibiting Hsp90 with macbecin was the most potent activator of the HSR when Hsp70 availability was reduced, we conclude that Hsp90-dependent activation of the HSR occurs independently of Hsp70. These observations support a model where the HSR is activated by proteotoxic stress through Hsp90 and Hsp70 independently.

### Hsp90-dependent and -independent modes of activation are both major drivers of the HSR

**Figure 4.**
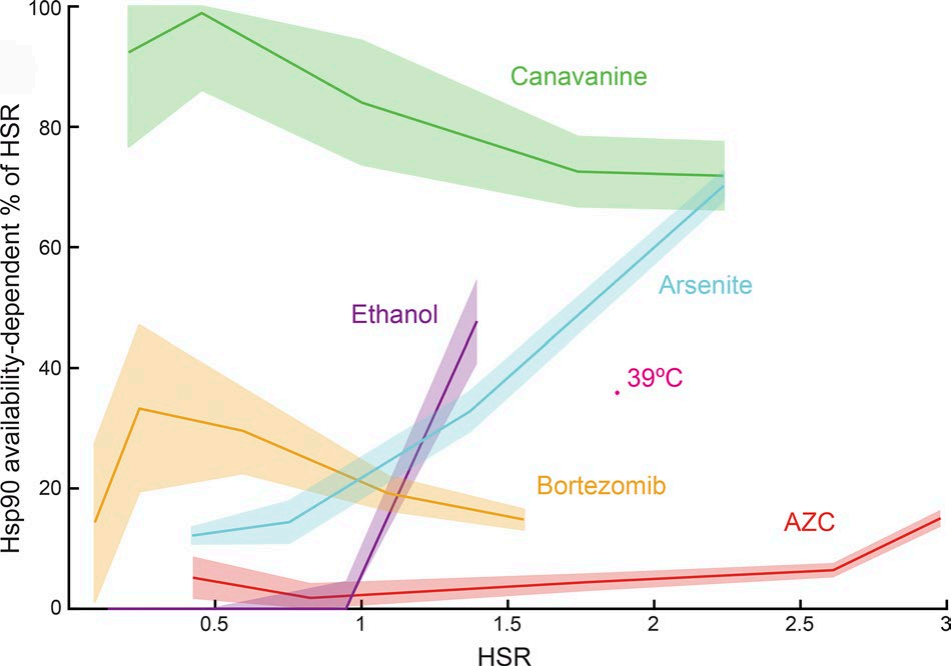
**Diverse mechanisms drive the HSR.** The percentage of Hsp90 availability-dependent HSR is plotted versus the total HSR over the course of each stressor titration. The HSR attributable to Hsp90 availability-dependent activation was calculated based on the assumption that the macbecin titration (a direct inhibitor of Hsp90) caused completely Hsp90-dependent HSR activation. *n* > 3; mean ± SEM.

Our finding that the HSR is regulated independently by Hsp90-dependent and -independent pathways allowed quantification of the underlying pathways driving the HSR for each environmental stress. Using a multiplicative model for interaction between activation pathways that has been employed to model genetic interactions of the HSR (Brandman et al., 2012), we deconvolved the HSR into Hsp90-dependent and -independent pathways (Fig. 4). Both Hsp90-dependent and Hsp90-independent pathways were major regulators of the HSR, depending on the strength and character of the stressor. Assumptions of this model are that the fluorescence measurements (made 5.5 h after stressor addition) are steady-state measurements and that changes in the Hsp90 availability reporter activity reflect changes in Hsp90 availability and not other cellular factors. This represents the first quantification of Hsp90 availability and its effects on the HSR in an in vivo context.

A critical feature of the HSR is that it is able to sense a diversity of stressors. How this occurs has been a topic of active debate and has implications for understanding and treating disease. Our work shows that the HSR integrates information from multiple chaperones and thus responds to a multidimensional view of the protein-folding landscape. These mechanisms, combined with other activation mechanisms responding to signaling pathways (Hahn and Thiele, 2004; Wang et al., 2006; Gomez-Pastor et al., 2017), suggest that HSR has the potential to act as a “computer” that integrates multiple stress inputs into a protective response. This paradigm highlights the complexity of the HSR and suggests that quantitative approaches that monitor multiple cellular systems such as those employed in this study will be instrumental in making further advances. We speculate that these approaches will be useful in understanding the complex and poorly understood protein quality control failures associated with neurodegenerative disease and aging as well as identifying therapeutic strategies for modulating the HSR.

## Materials and methods

### Reagents

Stock solutions were made for each of the stresses used in this experiment. Macbecin I (Tocris Bioscience) was dissolved at 10 mM in DMSO. L-Can (Sigma-Aldrich) and (S)-(−)-AZC (Alfa Aesar) were dissolved at 500 mM in water. Bortezomib (Sigma-Aldrich) was dissolved at 5 mM in ethanol. Sodium (meta)arsenite (Sigma-Aldrich) was dissolved at 100 mM in water. Ethanol (Gold Shield Distributors) was used pure. Additional chemical stocks used were cycloheximide (Sigma-Aldrich) dissolved at 50 mg/ml in ethanol and 21HP (Sigma-Aldrich) dissolved at 2 mg/ml in ethanol.

### Yeast strain construction and culturing methods

All experiments were done using the same parent strain of *S. cerevisiae* strain BY4741. Knockouts (see Table S2) were made through PCR of the NATMX6 or KANMX6 with 40-bp overhangs (see Table S3). Yeast were then grown to log phase (OD_600_ of 0.4–0.6), pelleted, washed twice in milliQ water, and resuspended in a solution containing 15 µl unpurified PCR product and transformation solution (120 µl of 50% polyethylene glycol 3.35K, 18 µl of 1 M lithium acetate, and 25 µl boiled salmon sperm DNA). The yeast were then incubated at 30°C for 30 min, incubated at 42°C for 20 min, and then centrifuged and allowed to recover overnight in 1 ml YPD. Yeast were then plated on YPD plates containing the appropriate drug selection. Colonies were further checked for integration of the cassette at the correct location through PCR.

The heat shock reporter is an integrated 4×HSE synthetic promoter upstream of an Emerald GFP as described previously (Brandman et al., 2012). The pUAS-GFP and pTDH3-GFP plasmids are identical to the heat shock reporter except that they contain either a UAS synthetic promoter on a 2µ plasmid or 644 bp TDH3 promoter on a Cen/Ars plasmid, respectively. The pL2/GG plasmid was created through replacement of the β-galactosidase gene of the pL2/GZ with GFP via restriction enzyme–based cloning. Transformations were done through the same heat shock protocol described above for yeast knockouts except that 100 ng plasmid was used instead of PCR product, and yeast were not recovered overnight and were instead plated on synthetic defined media lacking the appropriate amino acids to maintain selection (see Table S1).

Yeast containing these plasmids were grown from a single colony overnight at 30°C in synthetic defined media lacking the appropriate amino acids to maintain selection. Additionally, yeast containing the pUAS-GFP plasmid were grown overnight in synthetic media containing 2% raffinose instead of 2% glucose. Yeast were then rediluted in fresh media of the same type to ∼0.05 OD for subsequent growth. All fluorescence measurements were made with yeast in log phase with an OD_600_ of <0.7.

### Fluorescent reporter assay measurements

For the titrations of stress compounds, two strains of yeast were used: BY4741 yeast containing the Hsp90 availability reporter (pHCA/N795GR and pL2/GG plasmids) and BY4741 yeast containing the HSR reporter (4×HSE) and nonfluorescent versions of the Hsp90 availability reporter (pHCA/N795GR and pL2/GZ plasmids). The yeast were diluted such that after overnight growth (>10 h), they were in mid-log phase. After overnight growth, the yeast were diluted into a 96-well plate containing the stressor at a titration of concentrations. Four wells of yeast were grown per drug concentration. The yeast were grown for 3 h at 30°C while shaking at 1,300 rpm with the indicated drug (or at 39°C without any drug in the case of the heat shock stressor). Subsequently, 21HP was diluted from a stock of 6 mM (2 mg/ml; in ethanol) to 10 µM final concentration and was added to half the wells from both strains. Ethanol without 21HP was added to control wells (to 0.17% final concentration). After 2.5 h growth, cycloheximide was added to each well to a final concentration of 60 µg/ml to minimize changes in fluorescence during the measurement step. After 30 min in cycloheximide, GFP fluorescence was measured on a BD Accuri C6 flow cytometer (473 nm excitation; 510/15 filter). The titrations of stresses in the *hsc82Δ* and *ssa2Δ* strains (Fig. 4) was done in the same manner. The measurements of the knockout strains (Fig. 3) differed only in that overnight growth was to saturation. They were then rediluted to log phase in glass tubes and allowed to grow for 5 h (with no stressor) before addition of 21HP.

The experiments using the GFP-tagged Hsp90, pTDH3-GFP, and pUAS-GFP strains were all subjected to similar growth conditions and protocols but without addition of 21HP. Additionally, the pUAS-GFP strain was induced with 1% galactose 3 h after initial addition of the stressor (analogous to induction with 21HP for the Hsp90 availability reporter).

### Reporter quantification

Hsp90 availability was quantified based on the induction of GFP upon addition of glucocorticoid to the GR reporter (pL2/GG). The logarithm of GFP levels in the 21HP-induced state subtracted from the logarithm of GFP levels in the uninduced state. The base of the logarithm was chosen such that the logarithm of untreated WT yeast containing the Hsp90 availability reporter was equal to one. HSR levels were quantified as the base-2 logarithm of GFP levels in each cell minus the base-2 logarithm of GFP levels in a reference strain referred to in each figure. This reference strain was typically untreated WT yeast containing the HSR reporter.

To calculate the percentage of Hsp90-dependent HSR activation, we first decomposed the HSR into Hsp90-dependent and Hsp90-independent components. The Hsp90-dependent component of the HSR was computed based on the assumption that macbecin (an Hsp90-specific inhibitor) activation of the HSR was entirely Hsp90 dependent. A linear regression of the macbecin titration data was therefore used to create a model for the expected HSR for a given amount of Hsp90 availability. We then assumed a multiplicative model for Hsp90-dependent and Hsp90-independent HSR activation and computed the fraction of Hsp90-dependent HSR activation by dividing the logarithm of the Hsp90-dependent component of the HSR activation from the logarithm of the total HSR activation. Percentages were clipped to be in the range of 0% and 100% HSR activity.

### Cell viability measurements

Cell viability measurements were made following 6 h of drug treatment and 5 min of propidium iodide staining. Uptake of propidium iodide was measured on a BD Accuri C6 flow cytometer (552 nm excitation; 610/20 filter). We used cells treated with 50% ethanol as a positive control for cell death as well as untreated cells as a negative control for cell death. These controls were used to set a threshold level of propidium iodide for cell death in between these two populations. Fraction viability is calculated based on the ratio of cells below the set threshold compared with the total number of cells.

### Reverse transcription–quantitative PCR (qPCR) assay

Yeast containing the GR plasmid and Hsp90 availability reporter (pL2/GG) plasmid were grown as described above though in a larger volume (2 ml). These yeast were subsequently centrifuged at 7,000 *g*, and the pellets were flash frozen in liquid nitrogen. RNA was extracted from these yeast using an acid-phenol:chloroform (Thermo Fisher Scientific) extraction, and 10 µg RNA was DNase treated (Turbo DNase; Thermo Fisher Scientific) and then reverse transcribed (Multiscribe RTase; Thermo Fisher Scientific) using random hexamer primers. We then diluted the resulting cDNA fivefold in 2× SYBR green qPCR mix (Luna; New England Biolabs) and 500 nM primers to measure the C_t_ values for GFP and ACT1 in each of the conditions. GR induction was then measured as the ΔΔC_t_ of GFP using ACT1 as a loading control.

### Online supplemental material

Fig. S1 shows that proteotoxic stress treatments do not generally impair viability or protein expression in WT cells. Fig. S2 shows the effects of each proteotoxic stressor on Hsp90 levels. Fig. S3 shows that proteotoxic stress treatments do not generally impair viability or protein expression in hsc82Δ or ssa2Δ cells. Table S1 shows plasmids used in this study. Table S2 shows yeast strains used in this study. Table S3 shows primers used in this study.

## Supplementary Material

Supplemental Materials (PDF)

Tables S1-S3 (ZIP)
