# Supplementary material for: Quantification of Hsp90 availability reveals differential coupling to the heat shock response
Source: J Cell Biol. 2018 Nov 5;217(11):3809–16. doi: 10.1083/jcb.201803127 (PMC6219726; doi:10.1083/jcb.201803127)
Supplement: Supplemental Materials (PDF) [file JCB_201803127_sm.pdf]

Supplemental material

Alford and Brandman, https://doi.org/10.1083/jcb.201803127

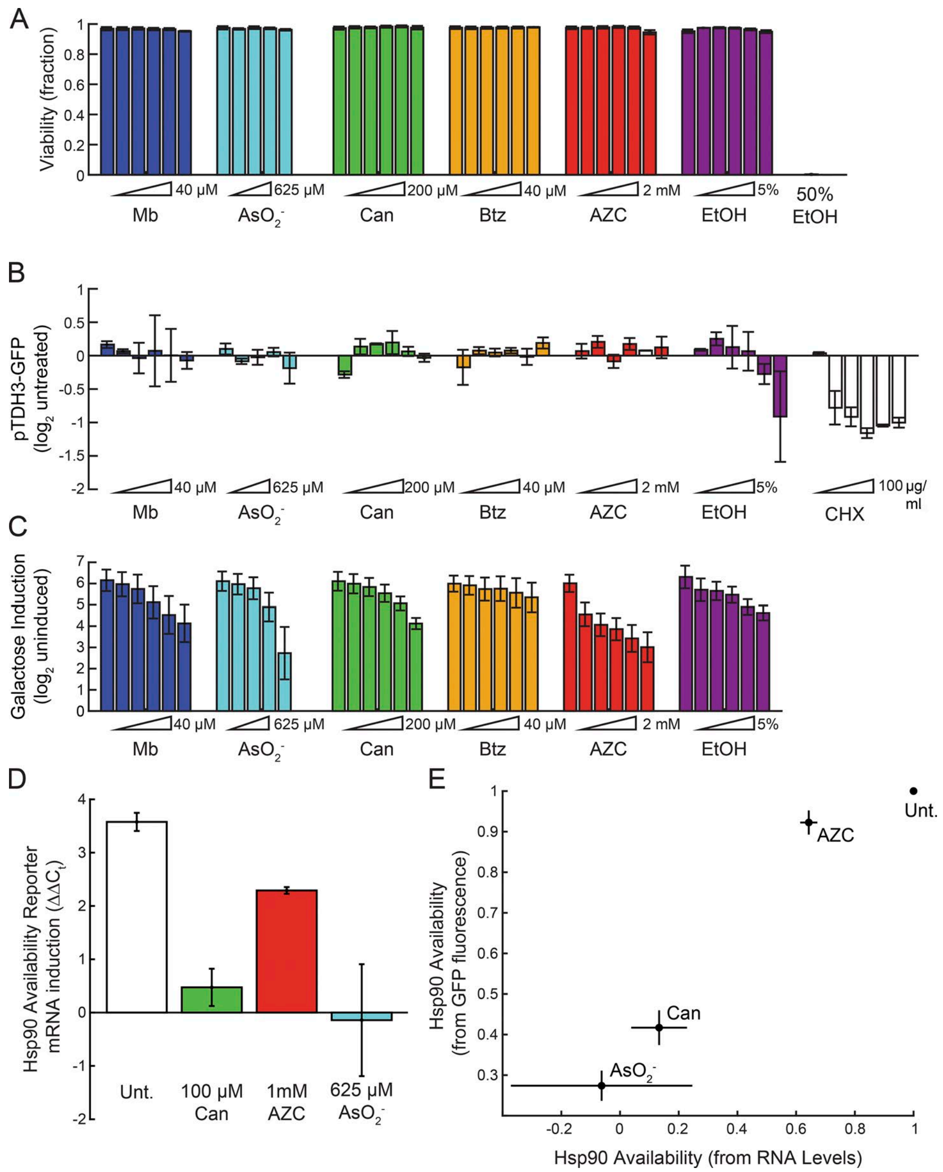

Figure S1. **Proteotoxic stress treatments do not generally impair viability or protein expression in WT cells. (A)** Fraction of cells that are viable (do not take up propidium iodide) under indicated treatments. **(B)** Expression levels of GFP under the control of the constitutive TDH3 promoter with indicated treatments. Cycloheximide treatment (CHX; white) was included as a positive control for inhibition of protein production. Values are normalized to no treatment (first bar of each color). **(C)** Galactose-induced GFP expression from a synthetic UAS promoter in the presence of indicated treatments. Fluorescence is normalized to the untreated (Unt.) condition. Btz, bortezomib; Mb, macbecin. **(D)** Hsp90 availability reporter mRNA induction as measured by qPCR of the GFP mRNA and normalized to ACT1 as a reference gene. **(E)** Hsp90 availability determined based on the changes in RNA levels versus Hsp90 availability calculated from GFP fluorescence. *n* = 3; mean ± SEM.

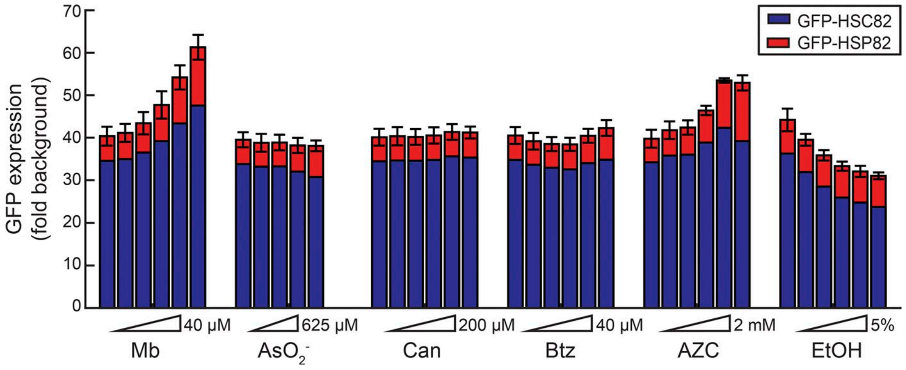

Figure S2. **Proteotoxic stressors have varying effects on Hsp90 levels.** A panel of stressors was added to two strains, each with one of the two Hsp90 genes (either *HSC82* or *HSP82*) tagged with a seamless, N-terminal GFP that preserved the endogenous promoter. The blue (lower) portion of the bar represents the fluorescence from the *GFP-HSC82* strain. The red (upper) portion of the bar is from the *GFP-HSP82* strain. The total height of the bar represents the total amount of Hsp90 in the cell. Btz, bortezomib; Mb, macbecin. *n* = 3; mean ± SEM.

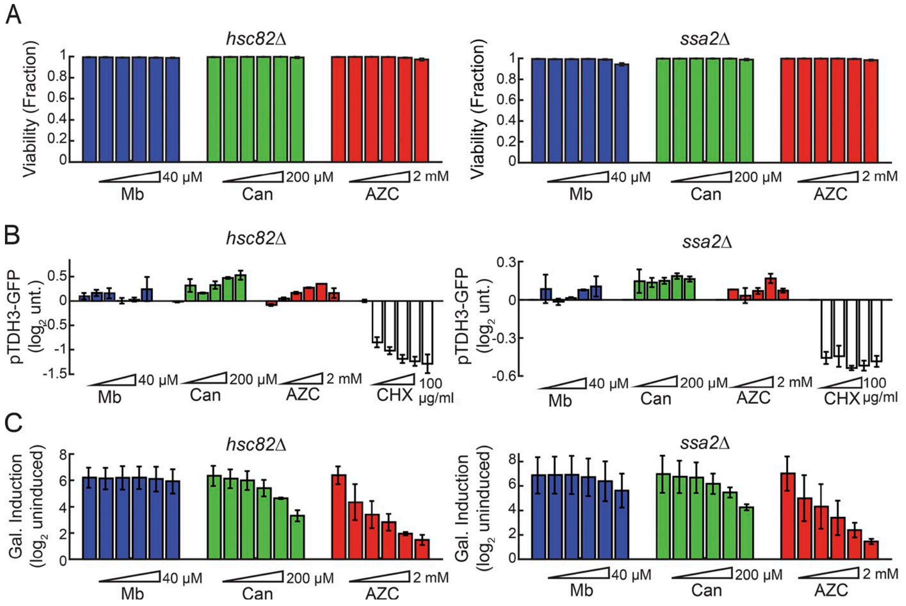

Figure S3. **Proteotoxic stress treatments do not generally impair viability or protein expression in hsc82Δ or ssa2Δ cells. (A)** Fraction of cells that are viable (do not take up propidium iodide) under indicated treatments (left, hsc82Δ; right, ssa2Δ). **(B)** Expression levels of GFP under the control of the constitutive TDH3 promoter with indicated treatments. Cycloheximide treatment (CHX; white) was included as a positive control for inhibition of protein production. Values are normalized to no treatment (first bar of each color). **(C)** Galactose-induced GFP expression from a synthetic UAS promoter in the presence of indicated treatments. Fluorescence is normalized to the uninduced condition (left, hsc82Δ; right, ssa2Δ). Mb, macbecin. *n* = 3; mean ± SEM.

Table S1 is a separate PDF showing plasmids used in this study.

Table S2 is a separate PDF showing yeast strains used in this study.

Table S3 is a separate PDF showing primers used in this study.
