# Supplementary material for: Quantification of Hsp90 availability reveals differential coupling to the heat shock response
Source: J Cell Biol. 2018 Nov 5;217(11):3809–16. doi: 10.1083/jcb.201803127 (PMC6219726; doi:10.1083/jcb.201803127)
Supplement: Tables S1-S3 (ZIP) [file JCB_201803127_TablesS1-S3.zip › JCB_201803127_TableS1.pdf]

Table S1: Plasmids

Plasmid	Description	Usage
pHCA/ N795GR[1]	HIS3, CEN/ARS, pTDH3-GR-N795	Rat GR; plasmid 1 of 2 for Hsp90 availability reporter; His selective
pL2/GG	LEU2, 2u, pGRE-GFP	GR-dependent synthetic promoter driving GFP expression; plasmid 2 of 2 for Hsp90 availability reporter; Leu selective
pL2/GZ[1]	LEU2, 2µ, pGRE-LacZ	GR-dependent synthetic promoter driving LacZ expression; used as a nonfluorescent control plasmid; Leu selective
pUAS-GFP	URA3, 2µ, pUAS-GFP	Galactose-inducible synthetic promoter driving GFP expression; Ura selective
pTDH3-GFP	URA3, Cen/Ars, pTDH3-GFP	Constitutive (TDH3) promoter driving GFP expression; Ura selective

[1]gifts from S. Lindquist (1281 and 1282; Addgene; Kimura et al., 1995)

Reference

Kimura, Y., I. Yahara, and S. Lindquist. 1995. Role of the protein chaperone YDJ1 in establishing Hsp90-mediated signal transduction pathways. *Science*. 268:1362–1365. https://doi.org/10.1126/science.7761857
