# Supplementary material for: Quantification of Hsp90 availability reveals differential coupling to the heat shock response
Source: J Cell Biol. 2018 Nov 5;217(11):3809–16. doi: 10.1083/jcb.201803127 (PMC6219726; doi:10.1083/jcb.201803127)
Supplement: Tables S1-S3 (ZIP) [file JCB_201803127_TablesS1-S3.zip › JCB_201803127_TableS2.pdf]

Table S2: Yeast Strains

Strain	Genotype	Description
yOB199	BY4741	wildtype strain
yOB255	BY4741 4xHSE-emGFP::URA3	HSR reporter
yJP384	BY4741 *hsc82Δ*::NAT	*hsc82Δ*
yOB445	BY4741 4xHSE-emGFP::URA3 *hsc82Δ*::NAT	HSR rep. in *hsc82Δ*
yJP385	BY4741 *ssa2Δ*::NAT	*ssa2Δ*
yOB446	BY4741 4xHSE-emGFP::URA3 *ssa2Δ*::NAT	HSR rep. in *ssa2*Δ
yB347	BY4741 *rqc1Δ*::NAT	*rqc1Δ*
yOB33	BY4741 4xHSE-emGFP::URA3 *rqc1Δ*::NAT	HSR rep. in *rqc1Δ*
yB259	BY4741 *ltn1Δ*::KAN	*ltn1Δ*
yB550	BY4741 4xHSE-emGFP::URA3 *ltn1Δ*::KAN	HSR rep. in *ltn1Δ*
yOB1608	BY4741 *get3Δ*::NAT	*get3Δ*
yOB1609	BY4741 4xHSE-emGFP::URA3 *get3Δ*::NAT	HSR rep. in *get3Δ*
yOB431	BY4741 *hsp104Δ*::NAT	*hsp104Δ*
yOB736	BY4741 4xHSE-emGFP::URA3 *hsp104Δ*::NAT	HSR rep. in *hsp104Δ*
yOB1565	BY4741 *Δsti1*::NAT	*sti1Δ*

yOB1566	BY4741 4xHSE-emGFP::URA3 *sti1Δ*::NAT	HSR rep. in *sti1Δ*
yOB1734	BY4741 *ypl225wΔ*::NAT	*ypl225wΔ*
yOB1735	BY4741 4xHSE-emGFP::URA3 *ypl225wΔ*::NAT	HSR rep. in *ypl225wΔ*
yOB1736	BY4741 *Δaim29*::NAT	*aim29Δ*
yOB1737	BY4741 4xHSE-emGFP::URA3 *aim29Δ*::NAT	HSR rep. in *aim29Δ*
yOB1738	BY4741 *hgh1Δ*::NAT	*hgh1Δ*
yOB1739	BY4741 4xHSE-emGFP::URA3 *hgh1Δ*::NAT	HSR rep. in *hgh1Δ*
yMS4344[1]	BY4742 can1Δ::GAL1pr-SceI::STE2pr-SpHIS5 lyp1 Δ::STE3pr-LEU2; HSC82pr-sfGFP-HSC82	Seamless GFP-tagged HSC82
yMS1588[1]	BY4742 can1Δ::GAL1pr-SceI::STE2pr-SpHIS5 lyp1 Δ::STE3pr-LEU2; HSP82pr-sfGFP-HSP82	Seamless GFP-tagged HSP82

[1]gift from M. Schuldiner (Yofe et al., 2016)

Reference

Yofe, I., U. Weill, M. Meurer, S. Chuartzman, E. Zalckvar, O. Goldman, S. Ben-Dor, C. Schütze, N. Wiedemann, M. Knop, et al. 2016. One library to make them all: streamlining the creation of yeast libraries via a SWAp-Tag strategy. *Nat. Methods*. 13:371–378. https://doi.org/10.1038/nmeth.3795
