# Supplementary material for: Quantification of Hsp90 availability reveals differential coupling to the heat shock response
Source: J Cell Biol. 2018 Nov 5;217(11):3809–16. doi: 10.1083/jcb.201803127 (PMC6219726; doi:10.1083/jcb.201803127)
Supplement: Tables S1-S3 (ZIP) [file JCB_201803127_TablesS1-S3.zip › JCB_201803127_TableS3.pdf]

Table S3. **Primers**

Primer	Sequence (5′–3′)
HSC82_MX_F	ACGCTACAGAACCAATAGAAAAATAGAATCATTCTGAAATgacatggaggcccagaatac
HSC82_MX_R	AATTTATATAATATATAAAACATGAAGGCGAAAAAAGAGAggatggcggcgttagtatc
SSA2_MX_F	CCAACAGATCAAGCAGATTTTATACAGAAATATTTATACAgacatggaggcccagaatac
SSA2_MX_R	AGTAAAACTTTTCGGATATTTTACAGGGCGATCGCTAAGCggatggcggcgttagtatc
RQC1_MX_F	GTCGTTGAATCATCTAAATATATTTGTAAAGTACTCGAACgacatggaggcccagaatac
RQC1_MX_R	CTTACTCTGCGTACTAGAAAATGAGCTGCAACATTTCTTAggatggcggcgttagtatc
LTN1_MX_F	CTAAGCCATCAAAAAAAGTTCAAGCAATAGTTGGTTCTTAgacatggaggcccagaatac
LTN1_MX_R	AAAAATGTAGTACATTTATATGAAATTTATATGCGATAGTggatggcggcgttagtatc
GET3_MX_F	AAACGTACGACAAGAACAAGAAGATCATCACATTGTAATTgacatggaggcccagaatac
GET3_MX_R	TTATATGTCGTATGTATCTATTTATGGTATTCAGGGGCTTggatggcggcgttagtatc
HSP104_MX_F	AAAGAAATCAACTACACGTACCATAAAATATACAGAATATgacatggaggcccagaatac
HSP104_MX_R	ATTCTTGTTCGAAAGTTTTTAAAAATCACACTATATTAAAggatggcggcgttagtatc
STI1_MX_F	TCCTCACTGTAGCTACTAAAACAACCTATACGCAAGAAAGgacatggaggcccagaatac
STI1_MX_R	AAAAGAATTCAAGATAATAAAGTTATATTTCGTATTATTTggatggcggcgttagtatc
YPL225W_MX_F	AGATGGACTAGAAGAACCACGACGAAACAATCATATAACAgacatggaggcccagaatac
YPL225W_MX_R	AATGTATTCTATATAATCAATCACAAATATATATGCATGTggatggcggcgttagtatc
AIM29_MX_F	TACCTCCCCCAAATTTTTTCTTGTTTGGTTGCATTATTTCgacatggaggcccagaatac
AIM29_MX_R	GCACTTACTTATTAATGAAGGCCATAAGCCAAACAACATCggatggcggcgttagtatc
HGH1_MX_F	AATTAAAAATAGCTCAAAAAAATCAACAAAAAAATTGAGTGgacatggaggcccagaatac
HGH1_MX_R	TATACAATTCTATGCTATGTGAACTGTCCTTGAAAGTGACggatggcggcgttagtatc

In the sequences, the capital region is the homologous region to the yeast genome that determines site of recombination (different for each primer set). The lowercase region is the part of the primer that binds to the MX cassette to amplify the selectable marker (same for each primer set).
